# Tailoring exceptional points with one-dimensional graphene-embedded photonic crystals

**DOI:** 10.1038/s41598-019-42092-2

**Published:** 2019-04-03

**Authors:** Shanshan Chen, Weixuan Zhang, Bing Yang, Tong Wu, Xiangdong Zhang

**Affiliations:** 1 Beijing Key Laboratory of Nanophotonics & Ultrafine Optoelectronic Systems, School of Physics, Beijing Institute of Technology, Beijing, 100081 China; 2 School of Physical Science and Information Engineering, Liaocheng University, Shandong, 252059 China; 3 Shandong Provincial Key Laboratory of Optical Communication Science and Technology, Shandong, 252059 China

## Abstract

We theoretically demonstrate that tunable exceptional points (EPs) can be realized by using graphene-embedded one-dimensional (1D) photonic crystals with optical pumping in the terahertz (THz) frequency range. By tuning the Fermi level of graphene sheet, the energy band are altered significantly and the EP appears. In particular, multiple EPs at different frequencies can be selectively produced via subtly adjusting the band structure. Furthermore, topological features of these EPs, such as crossing and anti-crossing of the real and imaginary parts of the eigenvalues, have been analyzed in detail. We expect that tunable EPs can provide an instructive method to design active optical devices based on photoexcited graphene sheets in the THz frequency range.

## Introduction

Since the pioneering work of Bender *et al*.^1^, it is recognized that the non-Hermitian Hamiltonian with parity-time (PT) symmetry can have purely real eigenvalues. Beyond some non-Hermiticity threshold, typically called the exceptional point (EP), the PT-symmetry is broken, and the system experiences an abrupt phase transition with eigenvalues becoming complex. The EP represents spectral singularity for the non-Hermitian Hamiltonian, where the eigenvalues and their corresponding eigenvectors coalesce simultaneously^2^. Inspired by the unique property, a lot of attentions have been paid to the PT-symmetric quantum systems^3–5^ and the concept of PT-symmetry has been successfully extended to wave optics. Photonic platforms are well-suited for constructing structures that satisfy the conditions of balanced gain and loss required by PT-symmetry^6–22^. In the PT-symmetric systems with EPs, a great variety of interesting optical phenomena have been discovered, such as asymmetric light propagation^8–10^ and invisibility^11,12^, Bloch oscillation of energy^13^, coherent perfect laser absorber^14–18^, single-mode laser^19,20^ and loss-induced suppression and revival of lasing^21,22^. While, the EP sustained in the previous investigation is fixed once the geometry and non-Hermitian parameters are determined. The EPs that can be tailored have many potential applications, such as controllable unidirectional invisibility and adjustable single-mode laser. However, the tunable EPs based on external fields are still rare.

On the other hand, due to the high-performance electric, thermal, mechanic and optical properties^23,24^, graphene has sparked keen interest and remained in the scientific limelight for over a decade, resulting in a rapid development of the field of graphene plasmonics. For example, the nanopatterned graphene sheet can be used as an active medium for infrared electro-optic devices^25,26^. Moreover, embedding graphene in the photonic crystal allows the system to exhibit desirable optical properties, such as enhanced nonlinear and absorption^27–31^. Furthermore, it have been demonstrated theoretically that loss induced amplification of graphene plasmons^32^ and singularity-enhanced sensing based on the PT-graphene metasurface^33^ are characteristics of EP behaviors. In addition, the utilization of the tunable graphene layer to control EPs has been rarely studied^34,35^. Motivated by the above investigations, the problem is whether or not the tunable EPs can be obtained in a quasi-PT symmetrical system, which is made up of the incorporation of graphene sheets into a one-dimensional photonic crystal with the PT symmetry.

In this work, we explore the possibility to tailor EPs in 1D graphene-embedded photonic crystals with optical pumping in the THz frequency range. By investigating the evolution of the complex band structures of the system, we find that the energy band can be effectively modified by tuning the Fermi level of graphene sheet and the EPs in THz frequency region emerge. Particularly, these EPs have topological features, such as the crossing and anti-crossing behaviors around them. In addition, many EPs at different frequencies are realized by altering only the Fermi level of the graphene sheet.

## Results and Discussions

### The graphene-embedded 1D photonic crystal and formulations of the model Hamiltonian

**Figure 1:**
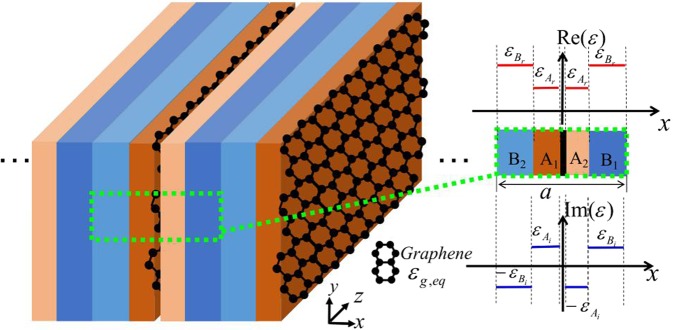
Schematic picture of a graphene-embedded photonic crystal and the profile of real/imaginary parts of the dielectric constants $[\mathrm{Re}(\varepsilon )/\text{Im}(\varepsilon )]$ in one unit cell.

The systems considered to realize the control of the EPs are 1D graphene-embedded photonic crystals, composed of the multi-layered unit cell that satisfies the quasi-PT symmetric condition with the tunable graphene layer. Here, we only show one of the systems we studied, and the other structures have similar results (see Supplemental Materials). As shown schematically in Fig. 1, each unit cell consists of five dielectric layers. The central layer (black) represents the photoexcited graphene sheet. The remaining four layers are gain (B_2_ and A_2_) and loss (B_1_ and A_1_) dielectrics with corresponding dielectric constants being ${\varepsilon }_{{B}_{2}}={\varepsilon }_{{B}_{r}}-i{\varepsilon }_{{B}_{i}}$, ${\varepsilon }_{{A}_{2}}={\varepsilon }_{{A}_{r}}-i{\varepsilon }_{{A}_{i}}$, ${\varepsilon }_{{B}_{1}}={\varepsilon }_{{B}_{r}}+i{\varepsilon }_{{B}_{i}}$, and ${\varepsilon }_{{A}_{1}}={\varepsilon }_{{A}_{r}}+i{\varepsilon }_{{A}_{i}}$, respectively. The thicknesses of A_1_ (A_2_) and B_1_ (B_2_) are taken to be d_A_ and d_B_. We can clearly figure out that the system presented in Fig. 1 is PT-symmetric, when the graphene sheets are neglected. While, the system degenerates into a general non-Hermitian system with graphene sheets being embedded in the original PT-symmetric system. Here the graphene sheet is considered as an extremely thin film, and the thickness is chosen to be ${\rm{\Delta }}=0.5\,nm$^36,37^. The optical properties of the graphene layer can be described by the equivalent permittivity *ε*_*g*,*eq*_, and the relationship between *ε*_*g*,*eq*_ and the complex surface conductivity of the graphene *σ*_*g*_ is expressed as^38^

1 $${{\rm{\varepsilon }}}_{g,\mathrm{eq}}={\rm{1}}+\frac{i{\sigma }_{g}{\eta }_{0}}{{{\rm{k}}}_{{\rm{0}}}{\rm{\Delta }}}$$

where ${\eta }_{0}(\,\approx \,377{\rm{\Omega }})$ is the impedance of air, ${k}_{0}=2\pi /\lambda$, *λ* is the wavelength of the incident wave in the air. The surface conductivity of the monolayer graphene consists of two parts: the intraband conductivity *σ*_int*ra*_ and the interband conductivity *σ*_int*er*_, which can be approximately expressed as (in the THz frequency)^39^:

2 $$\begin{array}{cc}{\sigma }_{\mathrm{int}ra}=\frac{2{e}^{2}{k}_{B}T\tau }{\pi {\hslash }^{2}(1+{\omega }^{2}{\tau }^{2})}\cdot \,\mathrm{log}(1+\exp (\frac{{E}_{f}}{{k}_{B}T}))+i\frac{2{e}^{2}{k}_{B}T\omega }{\pi {\hslash }^{2}({\omega }^{2}+1/{\tau }^{2})}\cdot  & \mathrm{log}(1+\exp (\frac{{E}_{f}}{{k}_{B}T}))\end{array}$$

3 $${\sigma }_{\mathrm{int}er}=\frac{{e}^{2}}{4\hslash }\cdot \,\tanh (\frac{\hslash \omega -2{E}_{f}}{4{k}_{B}T})+i\frac{{e}^{2}}{8\hslash \pi }\cdot \,\mathrm{log}(\frac{{(\hslash \omega +2{E}_{f})}^{2}}{{(\hslash \omega )}^{2}+{(2{k}_{B}T)}^{2}}),$$

where *e* represents the charge, *k*_*B*_ is the Boltzmann constant, $\hslash$ is the reduced Planck’s constant, $\omega =2\pi \nu$ is the angular frequency (*v* is the frequency of the incident wave), *τ* is the intra-band transition time, and *E*_*f*_ denotes the quasi-Fermi energy level of electrons and holes at temperature *T*. The energy splitting of the quasi-Fermi levels *E*_*f*_ is expressed as^40^

4 $${E}_{f}=6\alpha {(\frac{{\nu }_{F}}{{k}_{B}T})}^{2}\frac{\hslash {\tau }_{r}}{\pi \nu }\cdot I$$

where $\alpha \equiv {e}^{2}/4\pi {\varepsilon }_{0}\hslash c(\, \sim \,1/137)$ is the fine-structure constant, *v*_*F*_ is the Fermi-velocity of charge carriers in the graphene, *τ*_*r*_ is the recombination time for electron-hole pairs and *I* describes the intensity of the photo-doping pump source. It can be seen that *E*_f_ is highly tunable via the external pumping strength *I*, thereby affecting the optical properties of graphene. Motivated by the above characteristic, we study the evolution of the complex band structure by tuning the Fermi level of graphene sheet in such a general non-Hermitian system and explore whether the tunable EPs can be gained.

According to ref.^41^, in order to calculate the complex band structures of the non-Hermitian system, we should utilize the transfer matrix method to calculate the dispersion relation of the corresponding Hermitian part firstly, where the gain and loss of the photonic crystal are chosen to be zero. Then, the Hamiltonian for the non-Hermitian system can be set up by using the Bloch states of a fixed *k* (the Bloch wave vector) for the corresponding Hermitian part as a basis. The same method was used to investigate the band structure (TE mode) of the graphene-embedded 1D photonic crystal in this work.

Firstly, the eigen equation for the 1D photonic crystal in the absence of gain or loss can be obtained by using the transfer matrix method with the consideration of the boundary condition and the periodicity of the structure^42^. It can be expressed as:

5 $$T(\omega )(\begin{array}{c}{a}_{n}\\ {b}_{n}\end{array})={e}^{ika}(\begin{array}{c}{a}_{n}\\ {b}_{n}\end{array}),$$

where *a*_*n*_ and *b*_*n*_ are the amplitudes of the field propagating in the forward and backward directions in the medium layer marked with *n* (for the structure we studied, *n* is A_1_, A_2_, B_1_, B_2_ or Graphene), *k* is the Bloch wave vector, *a* is the lattice constant, and $T(\omega )$ is the product of the transmission matrix ${t}_{n\to n^{\prime} }$ and the propagation matrix *p*_*n*_ for the unit cell^43^. Here, $T(\omega )={p}_{{B}_{2}}{t}_{{B}_{2}\to {A}_{1}}{p}_{{A}_{1}}{t}_{{A}_{1}\to Graphene}{p}_{Graphene}{t}_{Graphene\to {A}_{2}}{p}_{{A}_{2}}{t}_{{A}_{2}\to {B}_{1}}{p}_{{B}_{1}}{t}_{{B}_{1}\to {B}_{2}}$. The dispersion relationship can be drawn and the gained Bloch states can be written as ${E}_{m}(x)={u}_{m}(x){e}^{ikx}$, where *u*_*m*_(*x*) is obtained from Eq. (5), *m* denotes the band index corresponding to any given *k*, which is a positive integer. Secondly, the Bloch wave functions of the non-Hermitian system can be described as ${\tilde{E}}_{l}(x)={\tilde{u}}_{l}(x){e}^{ikx}$ with ${\tilde{u}}_{l}(x)={\sum }_{l}{C}_{l,m}{u^{\prime} }_{m}(x)$, where $l=1,2,3,\cdot \cdot \cdot ,{l}_{\max }$, and ${u^{\prime} }_{m}(x)$ is the derivative function with normalized *u*_*m*_(*x*). Substituting this expansion into Helmholtz equation, we arrive at the corresponding model Hamiltonian for this non-Hermitian system:

6 $$\tilde{H}\tilde{C}={(\frac{\tilde{\omega }}{c})}^{2}\tilde{C},$$

where $\tilde{H}$ is a ${l}_{\max }\times {l}_{\max }$ matrix, $\tilde{C}$ is a column vector consisting of expansion coefficients *C*_*l,m*_, *c* is the speed of light in vacuum, $\tilde{\omega }$ is the angular frequency. From the above equations, the complex band structure of the non-Hermitian system can be calculated.

### The appearance of EP in graphene-embedded 1D photonic crystal

**Figure 2:**
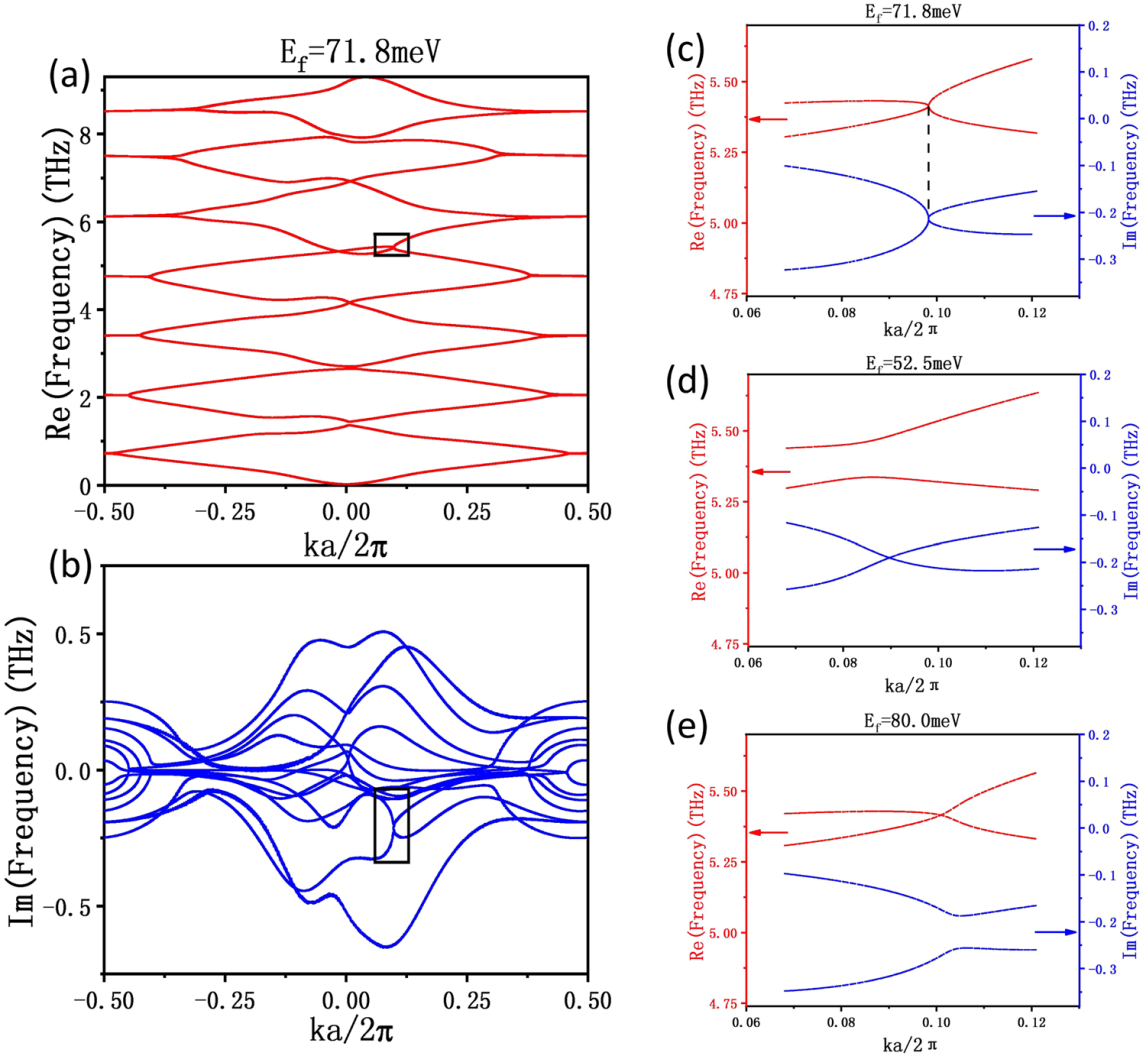
Real (**a**) and imaginary (**b**) parts of the complex band structures for the graphene-embedded 1D photonic crystal. The Fermi level of the graphene sheet is ${E}_{f}=71.8\,meV$. Other parameters of the system are chosen to be ${\varepsilon }_{{A}_{r}}=5.0$, ${\varepsilon }_{{B}_{r}}=7.8$, ${\varepsilon }_{{A}_{i}}={\varepsilon }_{{B}_{i}}=2.0$, ${d}_{A}=2.1\,\mu m$, ${d}_{B}=37.5\,\mu m$, and ${\mu }_{r}=1.0$. (**c**–**e**) Complex band structure in the black boxes of (**a**,**b**) with the fermi level being ${E}_{f}=71.8\,meV$, ${E}_{f}=52.5\,meV$ and ${E}_{f}=80.0\,meV$, respectively.

The eigenvalue of the above non-Hermitian system is generally complex, and the real and imaginary parts correspond to the energy and linewidth of the Bloch mode, respectively. The calculated complex band structures for the real and imaginary parts are depicted in Fig. 2(a,b), respectively. Here the Fermi level is $71.8\,meV$^44^, and other parameters are taken as: ${\varepsilon }_{{A}_{r}}=5.0$, ${\varepsilon }_{{B}_{r}}=7.8$, ${\varepsilon }_{{A}_{i}}={\varepsilon }_{{B}_{i}}=2.0$, ${d}_{A}=2.1\,\mu m$, ${d}_{B}=37.5\,\mu m$, ${\mu }_{r}=1.0$ and *l*_max_ = 25. As shown in Fig. 2(a,b), due to the existence of graphene sheet in the structure breaking the PT symmetry of the system, the dispersion curves of the considered structure are non-symmetric and the position with the smallest band gap is not necessarily formed at the center or boundary of the Brillouin zone. The black rectangular frames plotted in Fig. 2(a,b) mark the position of the EP, which deviates the center of the Brillouin zone. Therefore, the EPs may not be generated primarily at these high symmetry points, but may appear at other locations.

In order to clearly see the process of the emergence and disappearance of the EP, we present the complex band structures with different Fermi levels in Fig. 2(c–e). The red and blue curves within each figure represent the real and imaginary parts of the eigenfrequencies, respectively. The two curves have the same abscissa, and the red (blue) ordinate axis on the left (right) side is used to sketch the real (imaginary) part of the eigenvalues. Figure 2(c) shows the enlarged band structure marked in the box of Fig. 2(a,b), where the two curves intersect at the same abscissa ($ka/2\pi =0.0982$). That is to say, an eigenvalue whose real and imaginary part are equivalent simultaneously emerges and the EP is obtained. Such a phenomenon is very sensitive to the Fermi level of the graphene sheet. When *E*_*f*_ < *E*_*fEP*_ (*E*_*fEP*_ is the value for the appearance of the EP), such as ${E}_{f}=52.5\,meV$, red curves (real parts of eigenvalues) do not cross, and an intersection of blue curves (imaginary parts of eigenvalues) occurs, as shown in Fig. 2(d). While, when ${E}_{f}=80.0\,meV$ (*E*_*f*_ > *E*_*fEP*_), the red curves intersect, but the blue curves reopen.

From the above results, we find that the EPs can be gained by subtly adjusting the Fermi level of graphene sheet in the graphene-embedded 1D photonic crystal. It is very significant that the Fermi level can be controlled expediently by only tuning the external electric field. This is in contrast to the case without graphene, in which the EPs can only be obtained by changing the geometry (such as the thickness of the media) or dielectric parameters (such as loss and gain) of the system. Such a switching effect is very beneficial to the optical devices based on EPs.

### Topological structure of the EPs in the graphene-embedded photonic crystal

**Figure 3:**
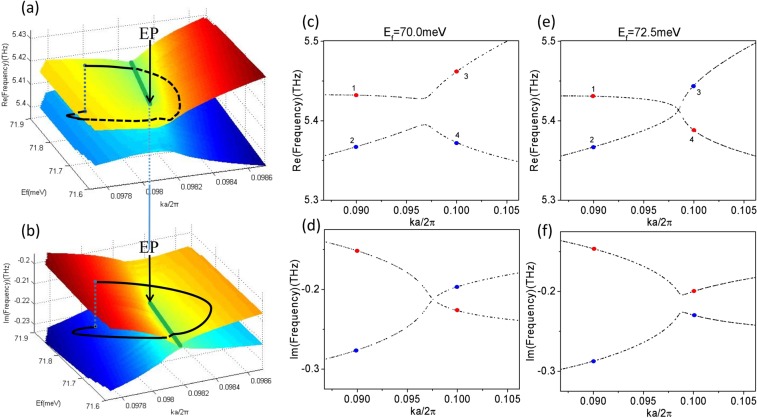
Perspective view of the Riemann sheet structure of two coalescing energy levels in the vicinity of the EP. Real (**a**) and imaginary (**b**) parts of the eigenvalues. The olive curves indicate the intersection of two surfaces. The black arrows point to the position of EP. The black curves represent the trajectory of the eigenvalues encircling the EP in the parameter space. The crossing and anti-crossing of the real and imaginary parts of the eigenvalues for *E*_*f*_ < *E*_*fEP*_ and *E*_*f*_ > *E*_*fEP*_ are shown in (**c**–**f**). All the other parameters are the same as shown in Fig. 2(a,b).

The results illuminated in Fig. 2(c–e) implies that the band structure has the characteristics of the Riemann surface. This can be clearly seen in Fig. 3. In Fig. 3(a,b), we plot the real and imaginary parts of the eigenvalues adjacent to the EP as functions of the Fermi level and normalized Bloch wave vector ($ka/2\pi$). We find that both two surfaces, belonging to the real and imaginary parts of the eigenvalues, intersect along the two olive curves. Furthermore, the two olive curves also intersect at a single point marked by black arrows. At this point, the complex eigenvalues coincide and an EP emerges.When encircling the EP in the parameter space constituted by the Fermi level of the graphene sheet and the normalized Bloch wave vector, the energy levels are exchanged. Consequently, the corresponding eigenmodes are also approximately exchanged. This EP have the topological features. The crossing and anti-crossing behaviors around it have been demonstrated.

We study the transition between crossing and anti-crossing by only changing the Fermi level of the graphene sheet. The real part of the complex band structures in the vicinity of the EP are plotted in Fig. 3(c,e) for ${E}_{f}=70.0\,meV$ and ${E}_{f}=72.5\,meV$, respectively. The corresponding imaginary parts are visualized in Fig. 3(d,f). One can clearly see the anti-crossing (crossing) behavior of the real (imaginary) parts of the complex eigenenergies in the photonic crystal with the smaller Fermi level (Fig. 3(c,d)) and the opposite behavior when the Fermi level increases to a value greater than *E*_*fEP*_ (Fig. 3(e,f)). In addition, comparing Fig. 3(c,e), the energy level at position 3 changes from the red level to the blue level, and at position 4 from the blue level to the red level.

### Tailoring EPs by tuning the Fermi level of graphene sheet

The above results only focus on the case with the EP formed around the 8th and 9th bands. In fact, many EPs emerged around other frequency ranges can also be obtained by precisely adjusting the Fermi level of the graphene sheet. In this part, we studied the lowest 12 bands of the 1D photonic crystal. Six EPs at different frequencies and normalized Bloch wave vectors are realized by altering the Fermi level of the graphene sheet.

**Figure 4:**
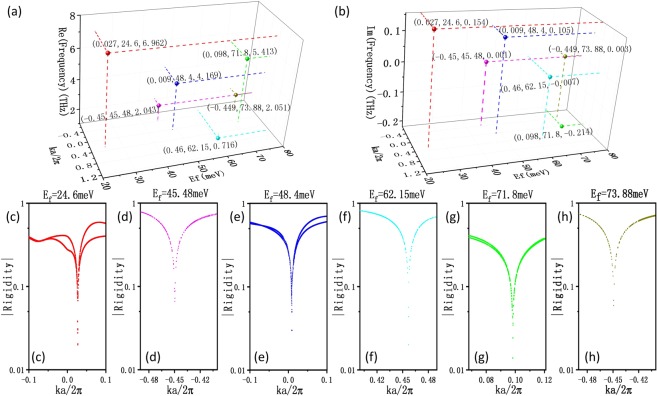
The normalized Bloch wave vectors, Fermi levels, and real (**a**) and imaginary (**b**) parts of the eigenfrequencies corresponding to the Eps we found. (**c**–**h**) The phase rigidities of the eigenstates obtained with different *E*_*f*_: ${E}_{f}=24.6\,meV$ (red dots), ${E}_{f}=45.48\,meV$ (magenta dots), ${E}_{f}=48.4\,meV$ (blue dots), ${E}_{f}=62.15\,meV$ (cyan dots), ${E}_{f}=71.8\,meV$ (green dots), ${E}_{f}=73.88\,meV$ (dark yellow dots), respectively.

The real and imaginary parts of the eigenfrequencies of different EPs are plotted in Fig. 4(a,b), respectively. The points with the same color correspond to the EP generated at a certain Fermi level of the graphene sheet. For example, the green dots represent the case with the Fermi level being ${E}_{f}=71.8\,meV$, that is the EP marked in Fig. 3(a,b). In this case, the normalized Bloch wave vector $ka/2\pi =0.098$, and the real part and imaginary part of the frequency are 5.413 and −0.214. Apart from this EP, the Fermi levels, normalized Bloch wave vectors, and real and imaginary parts of the eigenfrequencies corresponding to the other five EPs are ($24.6\,meV$, 0.027, $6.962\,THz$, $0.154\,THz$), ($45.48\,meV$, −0.45, $2.043\,THz$, $0.001\,THz$), ($48.4\,meV$, 0.009, $4.169\,THz$, $0.105\,THz$), ($62.15\,meV$, 0.46, $0.716\,THz$, $-\,0.007\,THz$), ($73.88\,meV$, −0.449, $2.051\,THz$, $0.003\,THz$), separately. These five EPs have the same properties as the EP mentioned above. We find that more than one EP emerge in the process of regulating the Fermi level, and they are distributed at different frequencies. This indicates that the switching between different EPs can be achieved in our system by just tuning the Fermi level of the graphene sheet. Consequently, even if the structure is determined, the EPs can also be tailored by using the external fields.

In order to further analyze the behavior of these EPs, we also calculate the phase rigidity. The phase rigidity is the quantitative measurement of the ratio between the orthogonality and the bi-orthogonality of the eigenstates, which has been discussed in detail in refs^45–47^. According to refs^46,47^, the phase rigidity is defined as:

7 $${r}_{l}=\frac{({{\tilde{u}}_{l}}^{L}(x)|{{\tilde{u}}_{l}}^{R}(x))}{\langle {{\tilde{u}}_{l}}^{R}(x)|{{\tilde{u}}_{l}}^{R}(x)\rangle },$$

where the parentheses on the numerator represent the bi-orthogonal product of the two terms and the denominator is the inner product of the two terms. The ${{\tilde{u}}_{l}}^{R}(x)$ represents the right eigenstate of the lth eigenvalue of the Hamiltonian and ${{\tilde{u}}_{l}}^{L}(x)$ is the corresponding left eigenstate. In non-Hermitian quantum mechanics, right and left eigenstates can be defined by the corresponding eigenvectors of the matrix representing the non-Hermitian operator in some complete set of orthonormal basis functions, which have been described in detail in ref.^48^. They can be expressed as:^48^

8 $${{\tilde{u}}_{l}}^{R}(x)=\sum _{m}{C}_{l,m}\sqrt{{\varepsilon }_{r}}{u^{\prime} }_{m}(x),$$

9 $${{\tilde{u}}_{l}}^{L}(x)=\sum _{m}{D}_{l,m}{(\sqrt{{\varepsilon }_{r}}{u^{\prime} }_{m}(x))}^{\ast },$$

where *D*_*l,m*_ is the element in the eigenvector $\tilde{D}$ of the transpose matrix of Hamiltonian $\tilde{H}$. The phase rigidity exhibits the degree of mixing of the two eigenstates near an EP and vanishes at the EP according to a power-law behavior^41^. During the process away from the EP, the phase rigidity gradually increases and is close to 1 at the position of the maximum width of the bifurcation. When the non-Hermiticity parameters are nonexistent, meaning that the system becomes Hermitian, the phase rigidity takes the maximum value of 1^45^. For the above 6 EPs, the corresponding phase rigidities are all calculated, as shown in Fig. 4(c–h). We find that the phase rigidities all vanish at these EPs. This further confirms that these points are actually EPs.

It is worthy to note that the above results are only for the photonic crystal with a certain geometry and non-Hermitian potential. In fact, if we change the thickness and the relative permittivity of the dielectric layer and keep the multi-layered unit cell satisfies the quasi-PT-symmetric condition, EPs can always be found by tuning the Fermi level of the graphene sheet. In addition, we want to point out that the geometric structure shown in Fig. 1 is not necessary to observe such a phenomenon. For the photonic crystals with the PT symmetry, when the graphene sheets are introduced in the systems, the structures that satisfy the quasi-PT-symmetric condition may be constructed. In Supplemental Materials, we provide the calculation results of tailoring exceptional points while using other geometric structures and parameters. Therefore, we can tailor EPs at any desired condition by reasonably designing the graphene-embedded 1D photonics crystal.

## Conclusions

In conclusion, using the non-Hermitian transfer matrix method based on the basis expansion with the results in the Hermitian potential, the band spectrum of a 1D graphene-embedded photonic crystal was obtained, exhibiting the EP in higher-order mode. The characteristics of the Riemann sheet structure and the phenomenon of crossing and anti-crossing of the eigenvalues adjacent to the EP were also studied. Furthermore, the behaviors of EPs have been disclosed by calculating the phase rigidity in the vicinity of the EPs. In particular, many EPs were gained at different frequencies by only tuning the Fermi level of graphene sheet. This means that we can tailor the EPs by using the external fields, which is very beneficial for the designs of optical functional devices based on the EPs.

## Supplementary information

sp
