# Supplementary material for: Tailoring exceptional points with one-dimensional graphene-embedded photonic crystals
Source: Sci Rep. 2019 Apr 3;9:5551. doi: 10.1038/s41598-019-42092-2 (PMC6447564; doi:10.1038/s41598-019-42092-2)
Supplement: Supplementary file 1 — sp [file 41598_2019_42092_MOESM1_ESM.pdf]

Supplemental Materials for

"Tailoring exceptional points with one-dimensional graphene-embedded photonic crystals"

Shanshan Chen[1], Weixuan Zhang[1], Bing Yang[1,2,3], Tong Wu[1], and Xiangdong Zhang[1]*

1 Beijing Key Laboratory of Nanophotonics & Ultrafine Optoelectronic Systems, School of Physics, Beijing Institute of Technology, Beijing, 100081, China

2 School of Physical Science and Information Engineering, Liaocheng University, Shandong 252059, China

3 Shandong Provincial Key Laboratory of Optical Communication Science and Technology, Shandong 252059, China

*Correspondence to zhangxd@bit.edu.cn

The calculation results of tailoring exceptional points while using other parameters or structures.

Here we provide the evidence that EPs can still be tailored by tuning the Fermi level of graphene sheet for other parameters. We still use the geometry shown in Figure 1, except that its corresponding parameters are taken as: $\varepsilon_{A_r} = 6.5$, $\varepsilon_{B_r} = 3.8$, $\varepsilon_{A_i} = \varepsilon_{B_i} = 1.5$, $d_A = 2.9\mu m$ and $d_B = 21.5\mu m$. As is shown in Figure S1, three EPs at different positions are realized by altering the Fermi level of the graphene sheet. The Fermi levels, normalized Bloch wave vectors, and real and imaginary parts of the eigenfrequencies corresponding to the EPs are ($11.45meV$, -0.448 , $1.509THz$, $0.002THz$), ($16meV$, -0.447 , $1.514THz$, $0.002THz$), ($33meV$, -0.236 , $12.051THz$,

$-0.011THz$) separately.

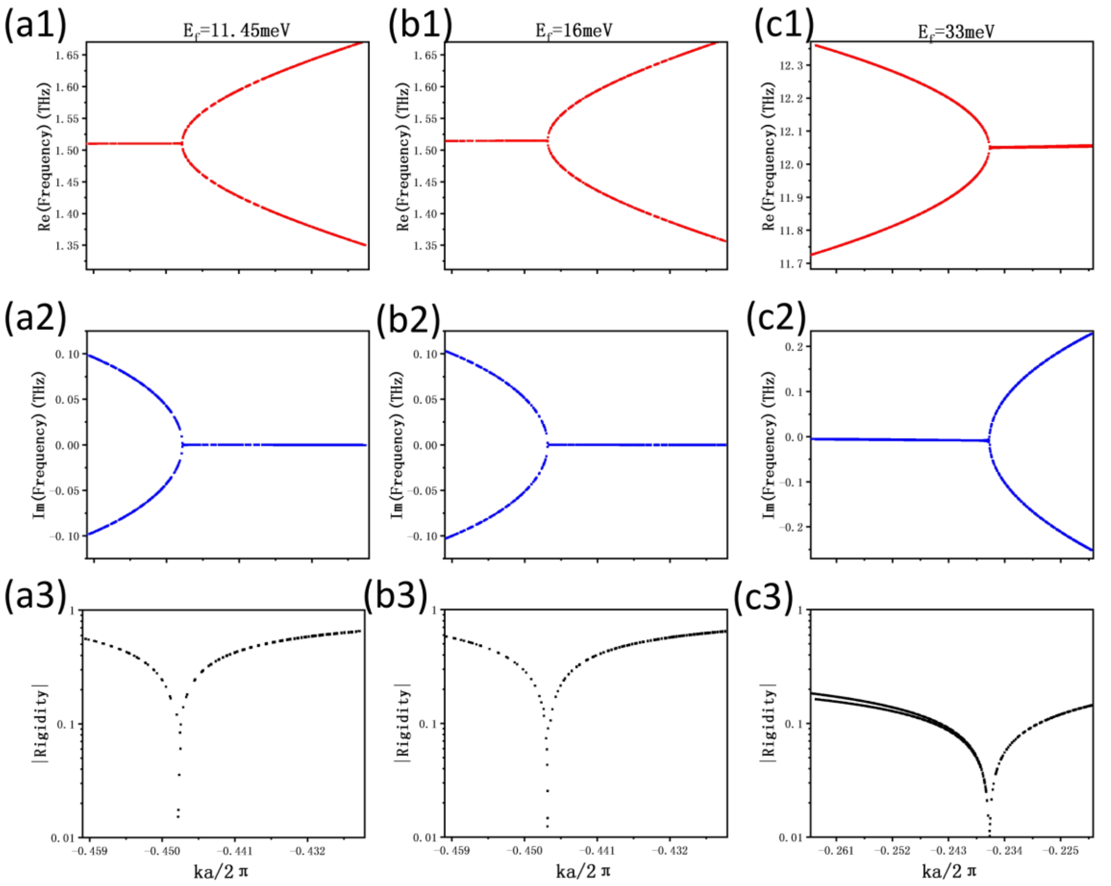

Figure S1. Real (a1,b1,c1) and imaginary (a2,b2,c2) parts of the complex band structures and the phase rigidities(a3,b3,c3) of the eigenstates obtained with different E_f: $E_f = 11.45meV$, $E_f = 16meV$ and $E_f = 33meV$. Other parameters of the system are chosen to be $\varepsilon_{A_r} = 6.5$, $\varepsilon_{B_r} = 3.8$, $\varepsilon_{A_i} = \varepsilon_{B_i} = 1.5$, $d_A = 2.9\mu m$, $d_B = 21.5\mu m$, and $\mu_r = 1.0$.

On the other hand, we have studied the EPs of a one-dimensional graphene-embedded photonic crystal with another geometric structure. As is shown in Figure S2, the unit cell of the system consists of six dielectric layers: B2, graphene, A1, A2, graphene and B1. The differences from Figure 1 are that this system has more than one layer of graphene in each unit, and the positions of graphene sheets have also

changed. The other parameters are identical. By tuning the Fermi level of graphene sheets, six EPs are obtained. The Fermi levels, normalized Bloch wave vectors, and real and imaginary parts of the eigenfrequencies corresponding to the EPs are ($11.25meV$, 0.337, $7.511THz$, $-0.005THz$), ($19.13meV$, -0.0157, $5.327THz$, $-0.113THz$), ($22.25meV$, 0.0121, $6.945THz$, $0.177THz$), ($33.3meV$, 0.457, $0.72THz$, $-0.007THz$), ($48.4meV$, 0.106, $5.427THz$, $-0.225THz$), ($92.3meV$, 0.354, $4.817THz$, $-0.02THz$), separately.

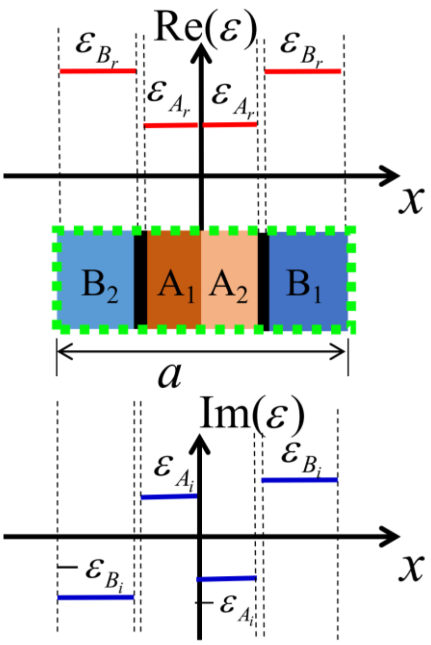

Figure S2. The profile of the one-dimensional graphene-embedded photonic crystal and real/imaginary parts of the dielectric constants $[\mathrm{Re}(\varepsilon)/\mathrm{Im}(\varepsilon)]$ in one unit cell.

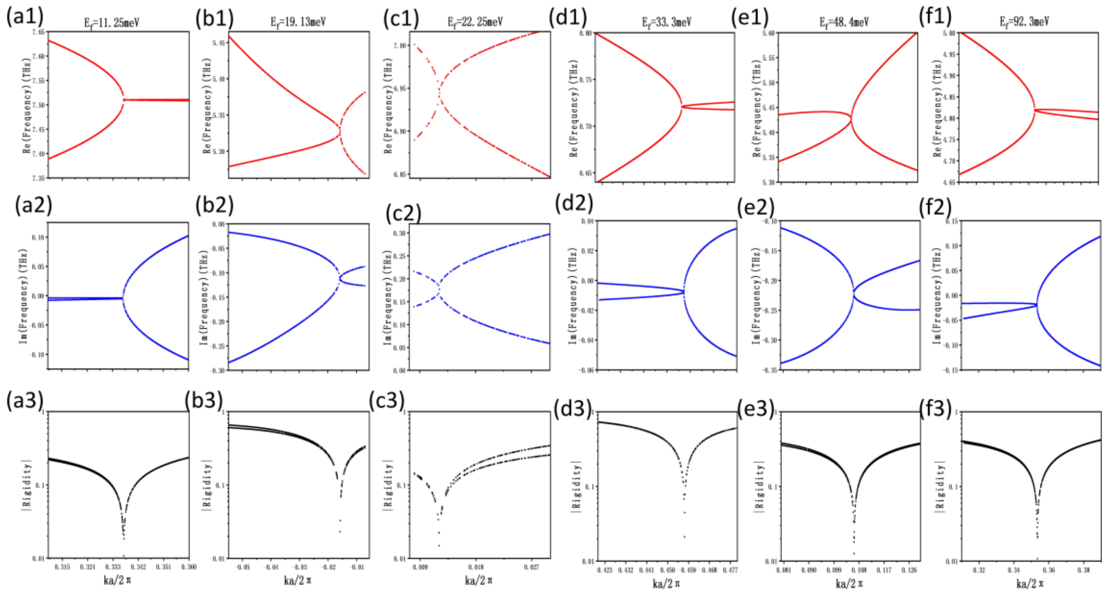

Figure S3. Real (a1,b1,c1,d1,e1,f1) and imaginary (a2,b2,c2,d2,e2,f2) parts of the complex band structures and the phase rigidities(a3,b3,c3,d3,e3,f3) of the eigenstates obtained with different E_f: $E_f = 11.25meV$, $E_f = 19.13meV$, $E_f = 22.25meV$, $E_f = 33.3meV$, $E_f = 48.4meV$ and $E_f = 92.3meV$. Other parameters of the system are chosen to be $\varepsilon_{A_r} = 5.0$, $\varepsilon_{B_r} = 7.8$, $\varepsilon_{A_i} = \varepsilon_{B_i} = 2.0$, $d_A = 2.1\mu m$, $d_B = 37.5\mu m$, and $\mu_r = 1.0$.
